# Supplementary figures and images for: Overexpression of Growth Differentiation Factor 15 in Glioblastoma Stem Cells Promotes Their Radioresistance
Source: Cancers (Basel). 2023 Dec 20;16(1):27. doi: 10.3390/cancers16010027 (PMC10778311; doi:10.3390/cancers16010027)

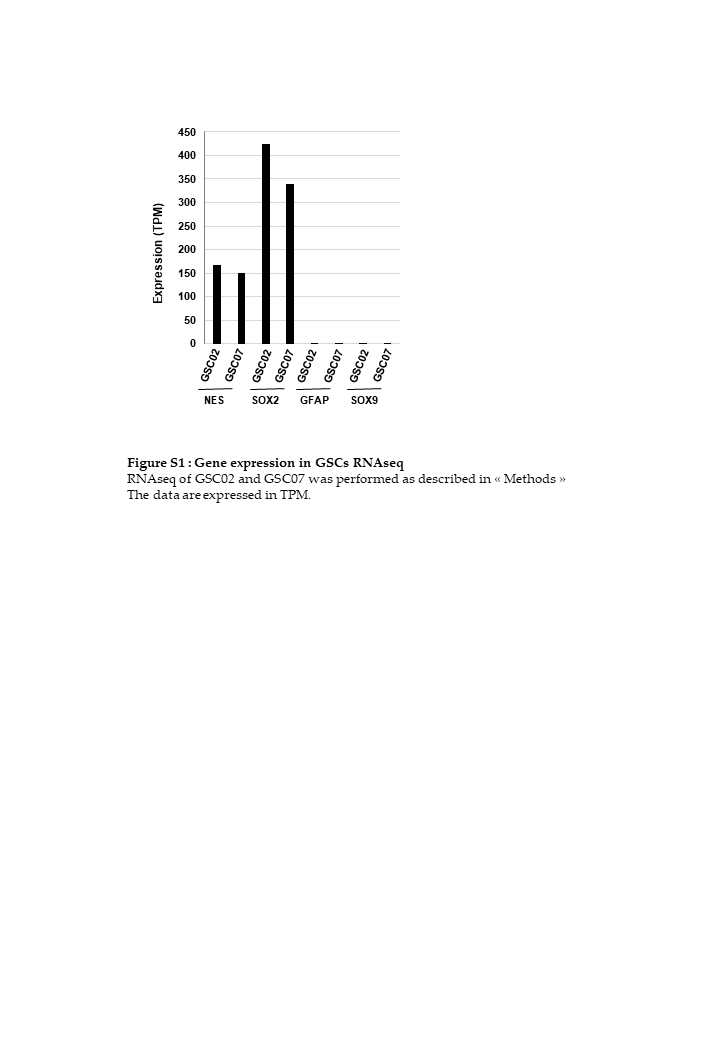

Supplement: Supplementary file 1 [file cancers-16-00027-s001.zip › supplementary material/figure S1.tif]

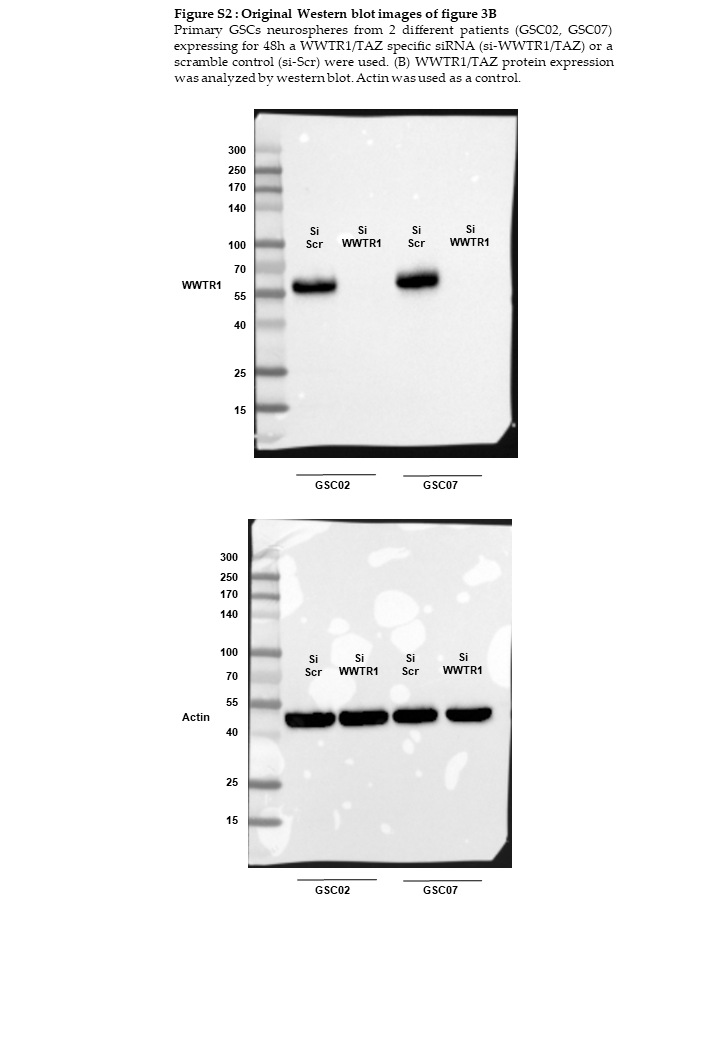

Supplement: Supplementary file 1 [file cancers-16-00027-s001.zip › supplementary material/figure S2.tif]

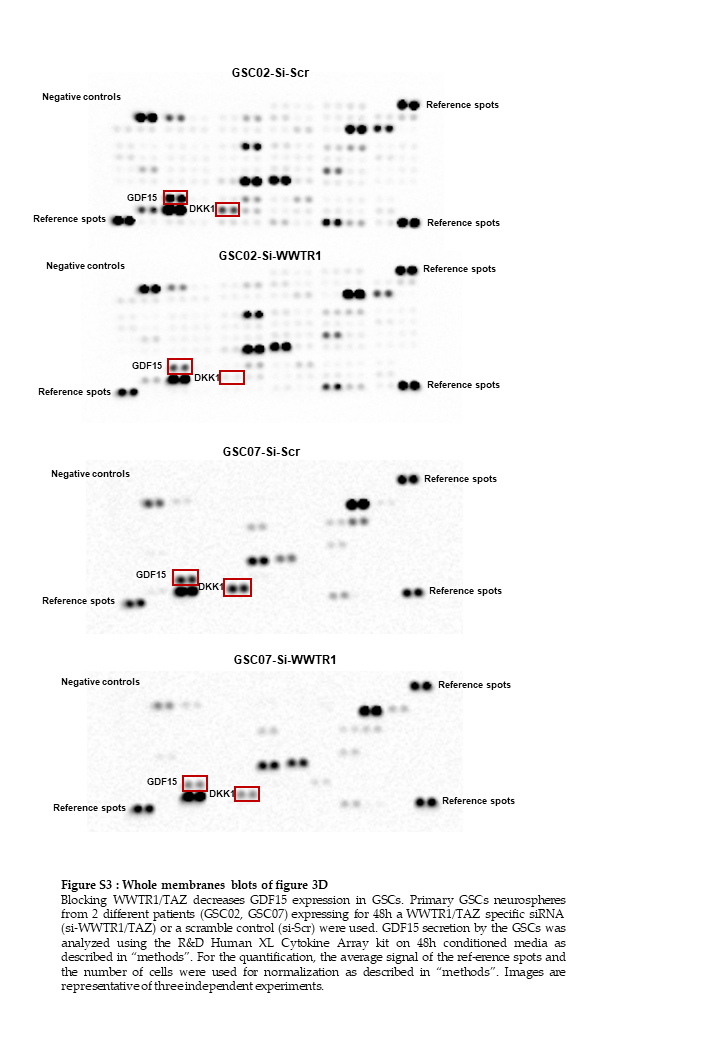

Supplement: Supplementary file 1 [file cancers-16-00027-s001.zip › supplementary material/figure S3.tif]

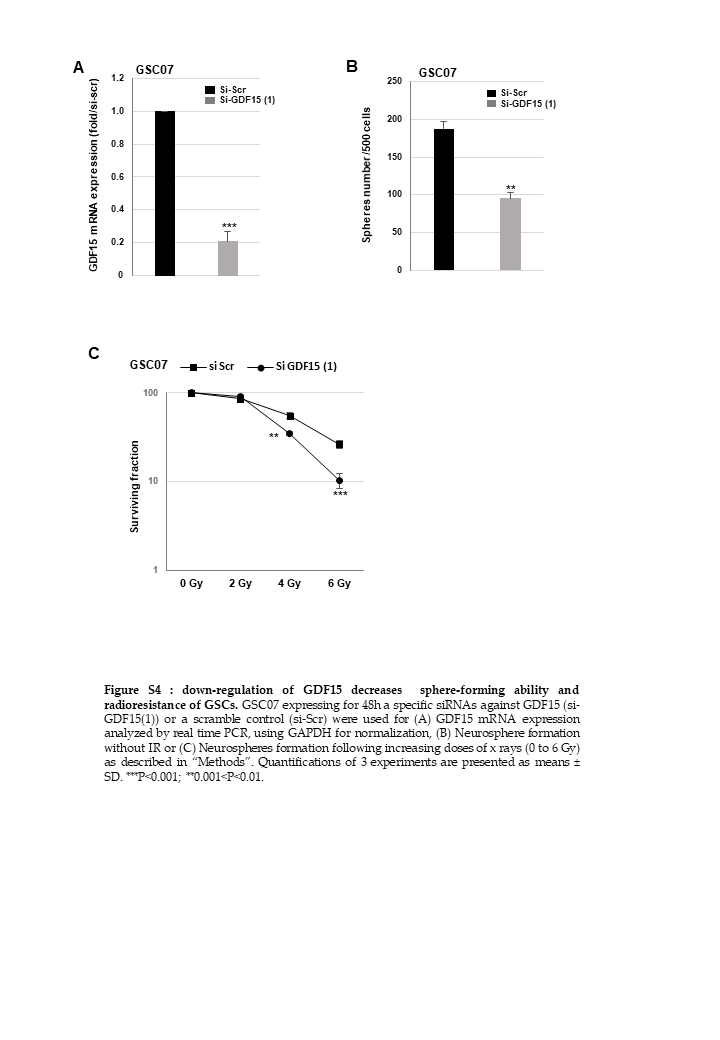

Supplement: Supplementary file 1 [file cancers-16-00027-s001.zip › supplementary material/figure S4.tif]

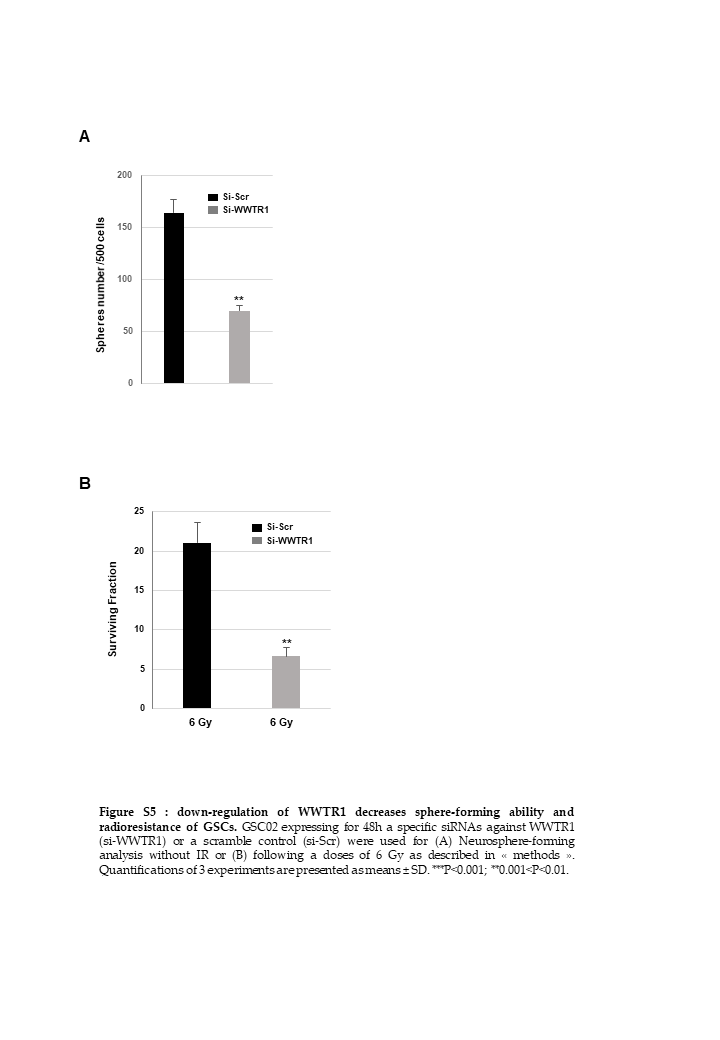

Supplement: Supplementary file 1 [file cancers-16-00027-s001.zip › supplementary material/figure S5.tif]
